# Debinding and Sintering of an Injection-Moulded Hypereutectic Al–Si Alloy

**DOI:** 10.3390/ma11050807

**Published:** 2018-05-16

**Authors:** Jiaqi Ni, Muhuo Yu, Keqing Han

**Affiliations:** State Key Laboratory for Modification of Chemical Fibers and Polymer Materials, College of Materials Science and Engineering, Donghua University, Shanghai 201620, China; jiaqini@mail.dhu.edu.cn (J.N.); yumuhuo@dhu.edu.cn (M.Y.)

**Keywords:** powder injection moulding, hypereutectic Al–Si alloy, debinding, sintering, microstructure, mechanical performance

## Abstract

Hypereutectic Al–Si (20 wt.%) alloy parts were fabricated by employing a powder injection moulding (PIM) technique with a developed multi-component binder system composed of high-density polyethylene (35 wt.%), carnauba wax (62 wt.%) and stearic acid (3 wt.%). The feedstocks contained 83 wt.% metal powders. The debinding process was carried out by a combination of solvent extraction and thermal decomposition. The effects of solvent debinding variables such as kind of solvents, debinding temperatures and time, and the bulk surface area to volume ratios on the debinding process were investigated. Thermal debinding and the subsequent sintering process were carried out in a heating sequence under a nitrogen atmosphere. The influences of sintering temperature and sintering time on the mechanical properties and structure were considered. Under the optimal sintering condition, sintering at 550 °C for 3 h, the final sintering parts were free of distortion and exhibited good mechanical properties. Relative sintered density, Brinell hardness, and tensile strength were ~95.5%, 58 HBW and ~154, respectively.

## 1. Introduction

Hypereutectic Al–Si alloys and their composites attract much attention for heat dissipation and electronic packaging applications due to their low density, high wear resistance, low thermal expansion coefficient and excellent thermal conductivity [1,2]. As electronic packaging strives continuously toward smaller size, higher integration and more complex geometries, conventional ingot metallurgy could not meet such requirements. Many efforts have been directed to the fabrication of hypereutectic Al–Si alloys and their composites using different process technologies. Sumitomo Electric Industries developed an Al (60 wt.%) and Si (40 wt.%) composite for electronic packaging by traditional powder metallurgy technology [3]. Hogg et al. [4] investigated the microstructure of a spray-formed Al–Si (30 wt.%) alloy used in electronic packaging applications. Zhang et al. [5] produced a 70 vol.% SiCp/Al–Si (12 wt.%) composite for electronic packaging using the pressure infiltration method.

Powder injection moulding (PIM) is a net shape manufacturing technology, which can mass-produce complex, precision, net-shape metal, ceramic or composite materials [6,7] and is a potential and attractive technology for the fabrication of miniature and complex metallic and ceramic package materials. PIM technology is a combination of conventional powder metallurgy technology and plastic injection moulding, which involves four main steps: feedstock preparation, moulding, debinding and sintering [6,8]. Feedstock preparation is conducted by blending solid powders and hybrid polymer binders together. The binders serve as a temporary phase to enhance the compressibility and fluidity of the fine powders and enable moulding to shape the desired geometry, using conventional plastic injection moulding technology. After the moulding process, the binders are removed from the moulded parts, and finally, the debinded parts are sintered to near full density.

There are several methods to remove the binders from the moulded parts, such as catalyst [9], wicking [10], solvents [11] and thermolysis [12]. Among them, the combination of the solvent and thermal debinding processes is widely used, due to the relatively high debinding efficiency, low cost of equipment and easy processing operation [13]. In the solvent debinding process, the soluble components of the binder are extracted to leave homogeneous porosity in the green parts, which improves removal of the residual components during thermal debinding. The types of solvent used, debinding temperatures and time, and the shape and size of green parts, influence the solvent debinding process.

The parts are sintered following the debinding process. Appropriate sintering conditions improve the structures of the sintered parts and ensure good mechanical properties [14,15]. Sintering of aluminum and its alloy is complicated because of the development of a thermodynamically stable oxide layer and its obstruction of inter-diffusion between powder particles and the shrinkage of pores between powder particles during sintering. Magnesium is much more chemically active than aluminum and can react with the oxide on the surface of aluminum powder to induce fracture and break-up of the thin oxide film and it, therefore, plays an important role in the sintering of aluminum and its alloys [16,17,18]. The atmosphere has a considerable influence on the sintering of aluminum and its alloys, and nitrogen is considered to be the optimal atmosphere [18,19]. Sn also is a beneficial activator for the enhanced liquid phase sintering of aluminum and its alloy. Trace amounts of Sn can improve the wetting characteristics and moderate the formation of aluminum nitride under nitrogen [20,21].

To satisfy the miniaturization, integration and mass production of electronic packaging materials, we developed an injection moulding process for hypereutectic Al–Si alloy. The objectives of the present study were to investigate the influences of solvent debinding variables on debinding processes and the effects of sintering parameters on the microstructure and properties of sintered parts. It was anticipated that the appropriate PIM process could expand the application of hypereutectic Al–Si alloy.

## 2. Materials and Methods

**Figure 1:**
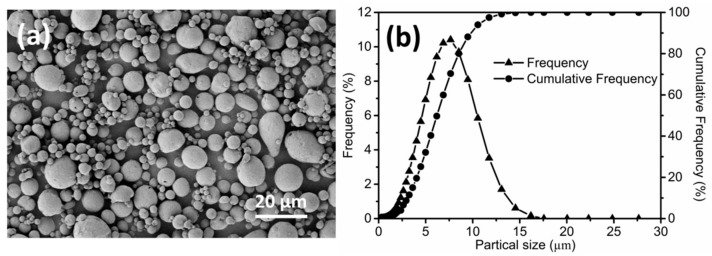
(**a**) Morphology and (**b**) granule size distribution of Al–Si alloy powders.

The morphology of Al–Si (20 wt.%) alloy powder and its granular size distribution, as determined using laser granulometry (BT-9300H, Bettersizer, Dandong, China), is shown in Figure 1. The Al–Si (20 wt.%) alloy particles were near-spherical and had a *D*_10_, *D*_50_ and *D*_90_ of 2.93, 5.73 and 9.76 µm, respectively. Minor amounts of pure Mg and Sn powders were used as sintering aids. The binder system consisted of 35 wt. % high density polyethylene (HDPE), 62 wt.% carnauba wax (CW), and 3 wt.% stearic acid (SA). The feedstock was prepared with a powder loading of 83 wt.% (80 wt.% Al–Si, 1 wt.% Mg and 2 wt.% Sn) based on our previous research.

**Figure 2:**
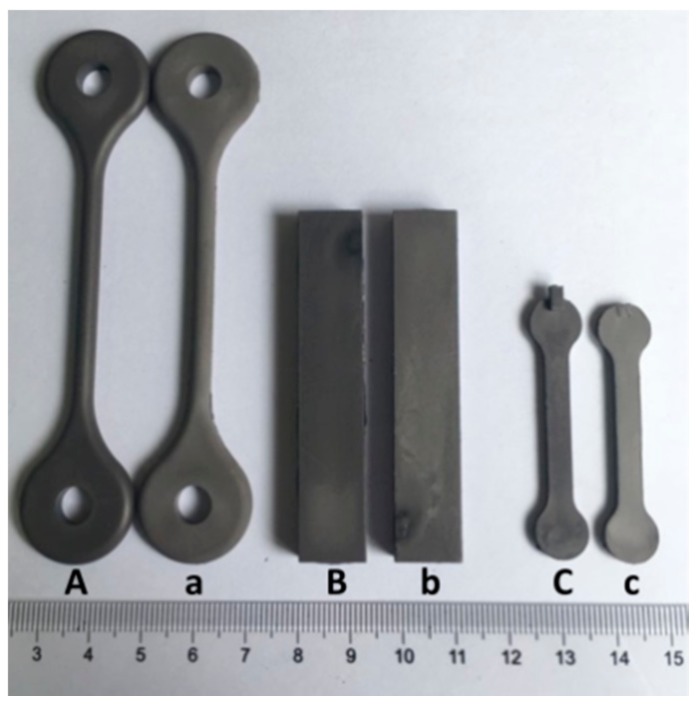
Demonstration of green parts (samples **A**–**C**) and solvent-debinded parts (samples **a**–**c**).

The feedstock was prepared using the hot solvent mixing method. The pre-mixed binder ingredients were added into a flask with xylene at 110 °C and stirred. After all the ingredients were dissolved completely, solid powder was added gradually to achieve the desired powder loading and continually stirred for 1 h. Finally, the xylene was distilled off and homogeneous feedstock was acquired. The injection moulding process was conducted using a reciprocating-screw type injection moulding machine (LX-MIM128, LASUM, Foshan, China). The green parts with three different shapes, shown in Figure 2 (samples A–C), were moulded by the machine. The bulk surface area to volume ratios (A/V) were about 0.677, 0.487 and 1.395 mm^−1^ respectively.

The elimination of binder was performed in two steps: solvent debinding to extract most of the soluble components (CW and SA) and thermal debinding to remove residual binders (HDPE and the little CW and/or SA). In the solvent debinding process, the green parts were immersed into organic solvents (xylene, hexane and heptane) for 1 to 23 h at different temperatures (50, 60 and 70 °C). The ratio of solvent to specimens was 10 mL/g. The parts were taken out from the solvents and vacuum-dried to evaporate the solvents after a predetermined interval to evaluate mass loss. In addition, to investigate the concentration effects of soluble components in the solvent, an experiment was performed in which the solvent was refreshed at predetermined intervals. Thermal debinding and sintering were performed in a tube furnace, which was filled with high-purity nitrogen and the gas flow was maintained at ~0.5 L/min. The heating sequence was established based on the thermal properties of the binder and its ingredients, as well as those of the Al–Si (20 wt.%) alloy powders. The effects of sintering temperature and sintering time on the properties of sintered parts were investigated.

The microstructure was examined using a scanning electron microscope (SEM, Quanta-250, Hillsboro, OR, USA) and an optical microscope (BX53M System Microscope, Olympus, Tokyo, Japan). Thermogravimetric analysis (TGA) and differential thermal analysis (DTA) were performed using a thermal analyzer (TA/Q5000IR, TA Instruments, Wymington, America) and a simultaneous thermal analysis system (STA409PC, NETZSCH, Selb, Germany), respectively, both at a heating rate of 10 min/°C in nitrogen. The density of sintered parts was measured using the Archimedes method. Tensile testing was conducted using an Instron 5969 machine (INSTRON, Canton, MA, USA) with a crosshead speed of 0.5 mm/min, utilizing specimens with a polished surface. The Brinell Hardness (HBW) was measured on a polished surface of the sintered parts, which were encapsulated in epoxy resin, using a 2.5 mm steel ball indenter and the load of 62.5 kgf. The phases of alloy powders and sintered parts were detected by X-ray diffraction (XRD, D/max-2550 PC, Rigaku, Tokyo, Japan) using a monochromatic target of Cu-Kα.

## 3. Results and Discussion

### 3.1. Solvent Debinding

**Figure 3:**
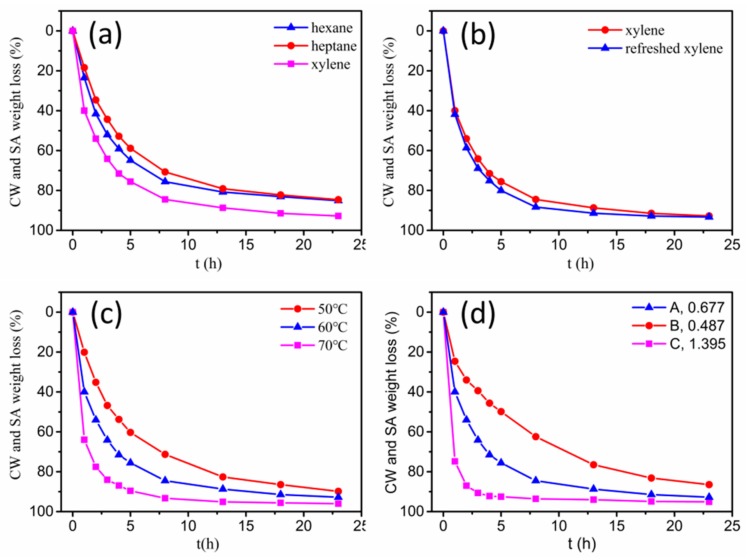
Carnauba wax (CW) and stearic acid (SA) weight loss (wt. %) of: (**a**) Specimens of sample A in different solvents at 60 °C; (**b**) Specimens of sample A in xylene with or without refreshing the solvent at each time-point at 60 °C; (**c**) Specimen of sample A in xylene at different temperatures; (**d**) Samples A, B and C in xylene at 60 °C.

**Figure 4:**
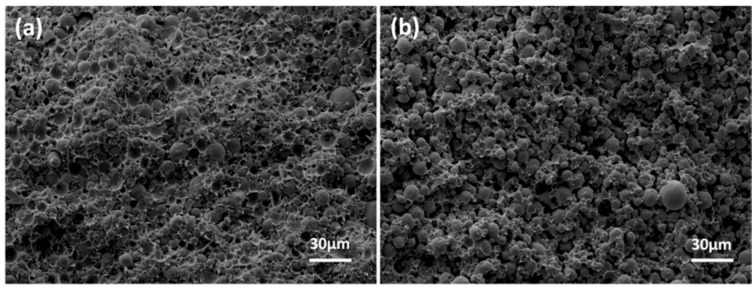
SEM micrographs of green parts C: (**a**) before and (**b**) after solvent debinding in xylene at 60 °C for 23 h.

The solvent debinding process of specimens in different solvents (hexane, heptane and xylene) at 60 °C is shown in Figure 3a. The xylene solvent had the highest extraction efficiency, as compared to hexane and heptane. It took ~6 h for xylene solvent to extract about 84.5 wt.% of the soluble components, while it took ~23 h in hexane and heptane. In addition, for all of the solvents, the debinding rate decreased as debinding time prolonged. The influence of refreshing xylene solvent on the debinding rate of specimens is shown in Figure 3b. At the initial stage of debinding, refreshing the solvent at each time-point promoted an improvement in extraction efficiency. While at a later stage, the weight losses in both situations tended to be consistent. It is well-known that solvent debinding is composed of two simultaneous processes: dissolution and diffusion. The results shown in Figure 3b implied that at initial stages of debinding, dissolution was the rate-determining step due to the direct contact of binder soluble ingredients with the solvent, which made the diffusion easier. Whereas, at a later stage, diffusion took the place of dissolution, becoming the rate determining step. Ultimately, both dissolution and diffusion proceeded with difficulty, and the solvent debinding process stabilized. The influence of temperature on the debinding process in xylene is shown in Figure 3c. A rise in extraction temperature promoted debinding efficiency, due to the influence of higher temperature on the solubility and diffusivity of CW and SA in xylene. The weight loss of binder soluble components from samples A, B and C after immersing in xylene at 60 °C as a function of time is shown in Figure 3d. As expected, specimens with different A/V values had different debinding progress. Samples with higher A/V values signified greater contact areas between the solvent and binder, and resulted in higher debinding rates. Solvent debinded parts (a–c) obtained from green parts (A–C) debinded in xylene at 60 °C for 23 h are shown in Figure 2. They were without any macro-defects such as cracks, distortion or bulges. Figure 4 shows the micromorphology of green parts and solvent debinded parts. It was observed that the alloy powders distributed homogeneously and were wrapped by the binder. There were no pores or inner cracks in the specimens. After solvent debinding, the specimen had homogeneous open pores, formed by the removal of the CW and SA. In addition, the brown parts had sufficient strength to be handled, which was attributed to the residual high density polyethylene (HDPE).

### 3.2. Thermal Debinding and Sintering

**Figure 5:**
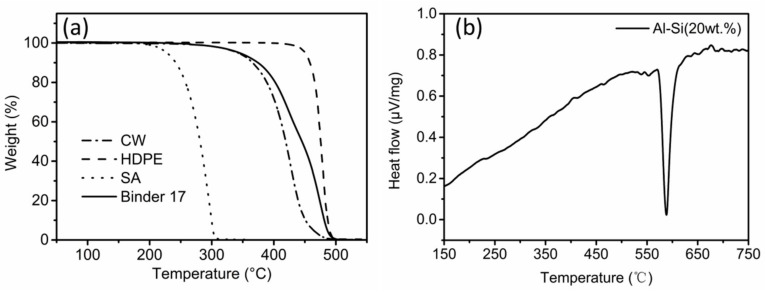
(**a**) TGA curves of binder and the pure ingredients; (**b**) DTA curve of Al–Si (20 wt.%) alloy powders.

The solvent debinded part, C, was chosen on which to perform the subsequent thermal debinding and sintering process. After solvent debinding, ~93 wt. % of the soluble ingredients were extracted. During the thermal debinding process, the remaining binders need to be removed. The thermal properties of binder and Al–Si (20 wt.%) alloy powders are shown in Figure 5. Figure 5a shows the thermogravimetric analysis (TGA) curves of the binder and its ingredients. CW/SA/HDPE started to degrade at 350/210/450 °C and evaporated completely at 500/315/500 °C, respectively. The thermal degradation of the binder was similar to the pure components, occurring in one step. It took place at about 200 °C and finished at approximately 500 °C. The gradual and wide decomposition temperature range was beneficial for thermal debinding [22]. In conclusion, up to 200 °C, as no decomposition took place, the heating rate was set 1 °C/min. From 200 to 500 °C, the remaining binders in the solvent-debinded samples decomposed and, as a slow heating rate can form homogeneous shrinkage and avoid bloating, blistering and other defects, a heating rate of 0.5 °C/min and hold time of 1 h at 500 °C was established.

DTA curves of Al–Si (20 wt.%) alloy powders heated from ambient temperature to 750 °C at a heating rate of 10 °C/min in nitrogen are shown in Figure 5b. An endothermic peak was observed only at 586.7 °C, corresponding to the melting of Al–Si (20 wt.%) alloy powder. A wide sintering temperature ranged from 520 to 600 °C during sintering for 1 h was investigated and sintering at 550 °C for different times was also performed. The complete thermal cycle of thermal debinding and sintering is summarized in Table 1.

**Figure 6:**
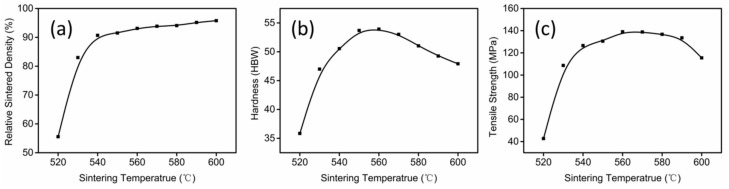
(**a**) Relative sintered density; (**b**) hardness; and (**c**) tensile strength of parts sintered at different sintering temperatures for 1 h.

The properties of sintered parts as a function of sintering temperature are shown in Figure 6. The results showed that the relative sintered density increased rapidly from 55.55% to 90.68% with rising temperature from 520 to 540 °C. Then, the density increased slowly with rising temperature from 540 to 600 °C. The Brinell hardness and tensile strength increased, with the sintered temperature increasing from 520 to 560 °C, and then decreased at different rates, respectively, as the sintering temperature increased continuously.

**Figure 7:**
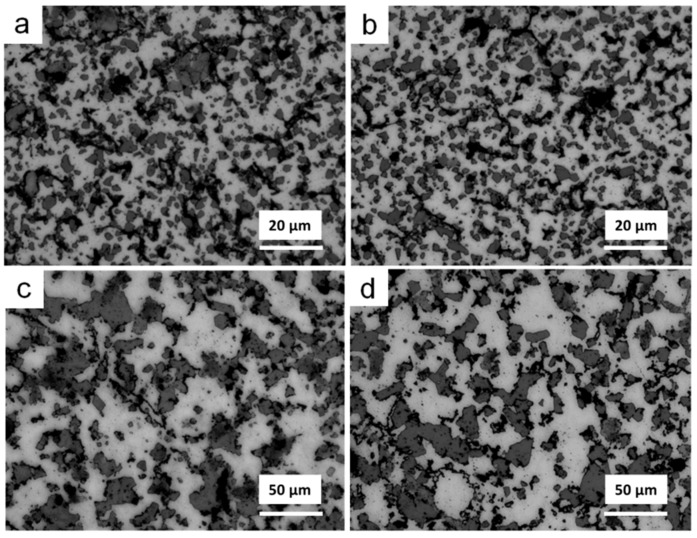
Optical microstructures of parts sintered at (**a**) 530 °C; (**b**) 550 °C; (**c**) 570 °C; and (**d**) 590 °C for 1 h.

Figure 7 shows the optical microstructures of parts sintered at different temperatures for 1 h. The primary Si of parts sintered at 530 °C (Figure 7a) and 550 °C (Figure 7b) was distributed homogeneously in the Al matrix and exhibited fine and irregular morphologies, with a size of less than 5 µm. Many microcracks formed between the primary Si particles or between the Si particles and the Al matrix. As sintering temperature increased, sintered at 570 °C (Figure 7c) or 590 °C (Figure 7d), the densification of sintered parts increased and microcracks reduced. However, the size of Si particles obviously increased up to 20 µm, and a few Si particles even had a size greater than 40 µm. The size increasing of primary Si seriously influenced the mechanical properties of the sintered parts.

**Figure 8:**
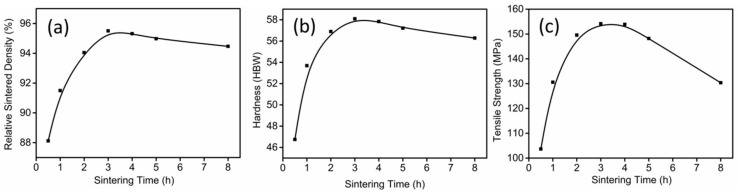
(**a**) Relative sintered density; (**b**) hardness; and (**c**) tensile strength of parts sintered at 550 °C for different sintering times.

The effects of sintering time on relative sintered density, hardness and tensile strength are shown in Figure 8. As expected, the relative sintered density increased with increasing sintering time and then maintained a steady value. The Brinell hardness and the tensile strength reached a maximum of ~58 HBW and ~154 MPa, respectively, after sintering at 550 °C for 3 h.

**Figure 9:**
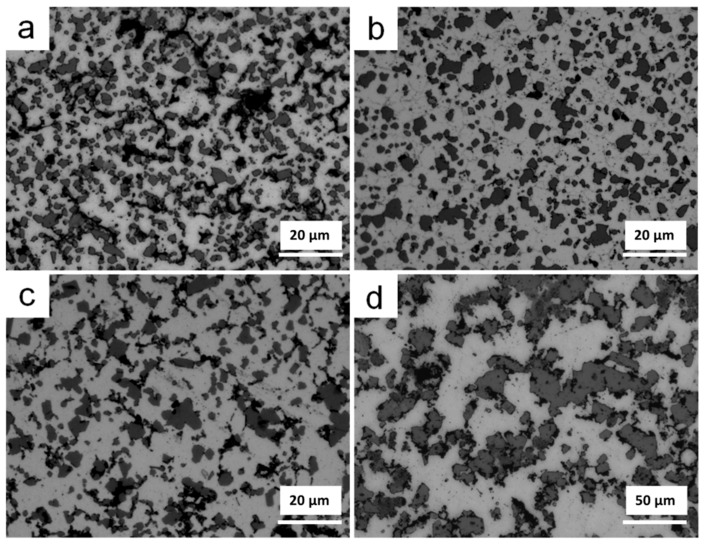
Optical microstructures of parts sintered at 550 °C for (**a**) 1 h; (**b**) 3 h, (**c**) 5 h and (**d**) 8 h.

Figure 9 presents the optical microstructures of parts sintered at 550 °C for different times. With an increase in sintering time, the size of the Si phase increased from less than 5 µm to greater than 25 µm, and the number of Si particles reduced substantially. The parts sintered at 550 °C for 3 h obtained optimal densification and had the best comprehensive mechanical properties. When sintering time was less than 3 h, the sintering reaction did not progress sufficiently, which resulted in many microcracks or holes in the sintered parts. Sintering for more than 3 h, the Si phase grew up, and was concentrated on the grain boundaries. The large size Si particles along the grain boundaries hindered the contact of grains, and led to the formation of defects and reduced density.

**Figure 10:**
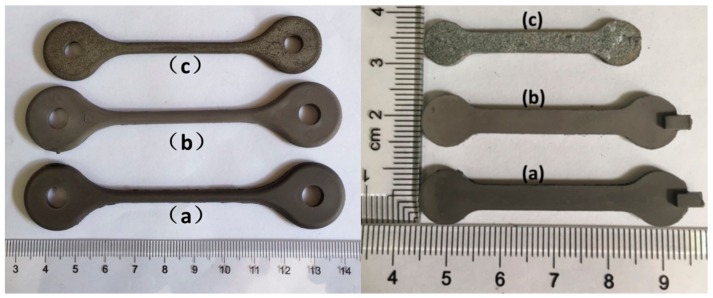
Shrinkage variation of sample A and C (**a**) green part; (**b**) solvent-debinded part; and (**c**) sintered part.

**Figure 11:**
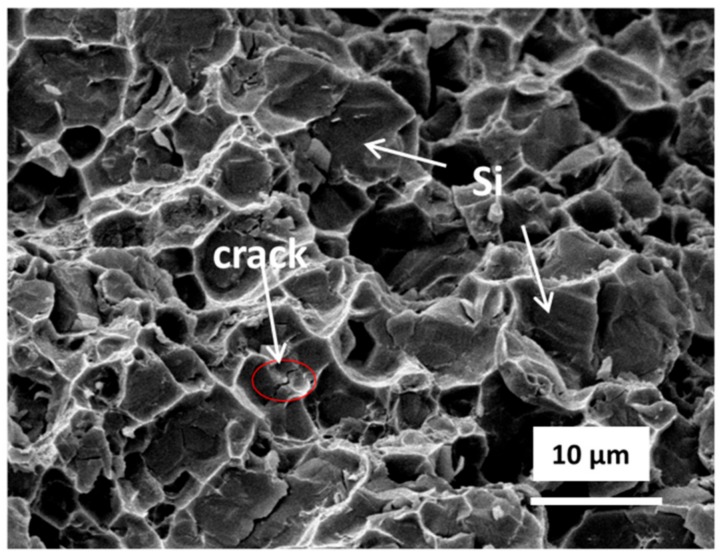
SEM image of tensile fracture surface of sintered part.

**Figure 12:**
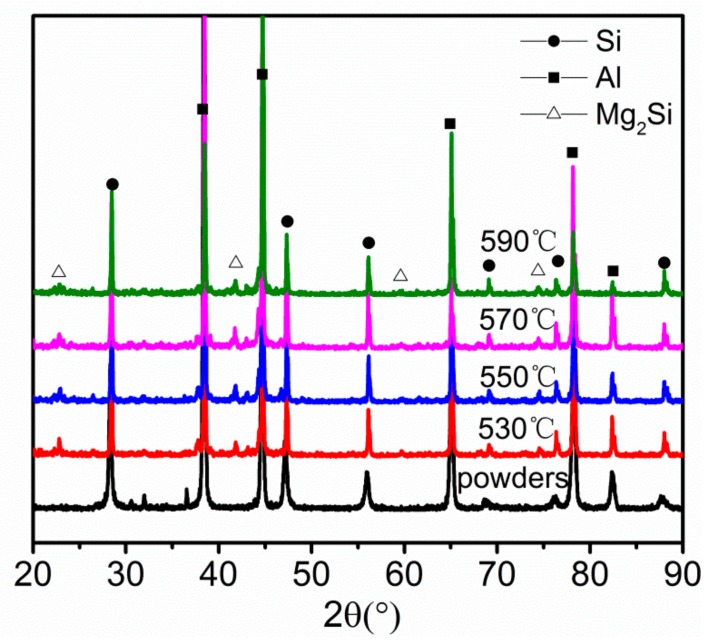
XRD patterns of alloy powders and sintered parts sintered at different temperatures.

Images of the green parts A and B, and their solvent debinded parts and sintered parts (sintered in nitrogen at 550 °C for 3 h) are shown in Figure 10. The sintered parts were free of distortion. There were no macroscopic defects such as cracks or blisters, and the shrinkage was uniform. Figure 11 shows the tensile fracture surface of sintered parts. As can be seen, the part exhibited some visible dimples, quasi-cleavage morphology and some cracks related to the Si particle. These indicated that the Al matrix failed via ductile rupture, and the Si phase failed via cleavage fracture. XRD patterns of alloy powders and sintered parts are provided in Figure 12. A comparison of the patterns of the alloy powders revealed that the additional peaks in the pattern of the sintered parts belonged to Mg_2_Si.

## 4. Conclusions

Hypereutectic Al–Si (20 wt.%) alloy parts were manufactured by powder injection moulding (PIM). Micron-sized hypereutectic Al–Si (20 wt.%) powder and a multi-component binder system consisting of HDPE, CW and SA were used to prepare the feedstocks. In the solvent debinding process, the influences of the kinds of solvents, debinding temperatures and time, and the bulk surface area to volume ratios on debinding rates were determined. Debinding in xylene had greater debinding efficiency than in hexane and heptane. The increment of debinding temperatures and the bulk surface area to volume ratios obviously decreased debinding time. Sintering temperature and time had a large influence on the microstructure and mechanical properties of the sintered parts. The final parts sintered at 550 °C for 3 h achieved a homogeneous microstructure and high-densification. The relative sintered density, Brinell hardness and tensile strength were ~95.5%, ~58 HBW and ~154 MPa, respectively.

## Figures and Tables

**Table 1:** Thermal debinding and sintering schedule.

Stage	Heating Rate (°C/min)	Debinding/Sintering Temperature (°C)	Hold Time (min)
1	1	ambient temperature to 200	0
2	0.5	200 to 500	1
3	0.8	500 to sintering temperature	Sintering time
4	furnace cooling	ambient temperature	0
